# Development and Validation of a New Method for Determination of Pb and Cr in Marine Organisms by Total Reflection X-Ray Fluorescence (TXRF) Spectroscopy

**DOI:** 10.1155/2019/8150678

**Published:** 2019-05-27

**Authors:** Blanca G. Beltrán, Ibiza Martínez-Serrano, Victor Ramos-Sanchez, David Chávez-Flores, Myrna C. Nevárez-Rodríguez, Emilio A. Suárez-Domínguez

**Affiliations:** ^1^Faculty of Nursing and Nutriology, Chihuahua Autonomous University, C.P. 31125 Chihuahua, Mexico; ^2^Museum of Zoology/Laboratory Hydrobiology, Faculty of Biology, Veracruzana University, C.P. 91090 Xalapa, Mexico; ^3^Faculty of Chemical Sciences, Chihuahua Autonomous University, C.P. 31125 Chihuahua, Mexico; ^4^Faculty of Agrotechnological Sciences, Chihuahua Autonomous University, C.P. 31125 Chihuahua, Mexico; ^5^Museum of Zoology, Faculty of Biology, Veracruzana University, C.P. 91090 Xalapa, Mexico

## Abstract

Lead and chromium contamination represents one of the most serious problems in the aquatic environments. The aim of this work was to develop and validate an accurate, sensitivity, and rapid method for the simultaneous determination of Pb and Cr at trace levels in tissues and fat of marine organisms such as turtle (*Chelonia mydas*), shark (*Rhizoprionodon terraenovae*), and dolphin (*Tursiops truncatus*), utilizing the total reflection X-Ray fluorescence (TXRF) spectroscopy. Working solutions were prepared in 10 mL of a solution 0.005 mol·L^−1^ EDTA and 1 mol·L^−1^ HNO_3_. In order to correct possible instrument drifts, 20 *μ*g·L^−1^ of gallium was used as internal standard (IS). The results showed that TXRF method was linear over the concentration ranges of 5.242–100 *μ*g·L^−1^ for Pb and 2.363–100 *μ*g·L^−1^ for Cr. Limits of detection (LOD) achieved were 1.573 and 0.709 *μ*g·L^−1^ for Pb and Cr, respectively, while limits of quantification achieved were 5.242 *μ*g·L^−1^ for Pb and 2.363 *μ*g·L^−1^ for Cr. The validated method was accurate and precise enough for determination of these heavy metals in samples of marine organisms as indicated by acceptable values of recovery between 90–101%. In addition, a certified reference material (BCR-279, sea lettuce) and a Centrum tablet were satisfactory analyzed, and the *T*-test for comparison of means revealed that there were no significant differences at the 95% confidence level between the values obtained with the proposed TXRF method and the certificated values. The repeatability of the method, expressed as relative standard deviation (RSD), was 5.1% and 4%, for Pb and Cr, respectively. In addition, other features of the developed method were a low sample volume of 10 *μ*L, and the sample frequency achieved was 20 h^−1^.

## 1. Introduction

The contamination of fresh and marine waters with a wide range of pollutants has become a matter of concern over the last few decades [1, 2]. Heavy metals are considered the most important form of pollution of the aquatic environment because of their toxicity and accumulation by marine organisms [3, 4]. The anthropogenic sources include mining effluents, industrial effluents, domestic effluents, urban storm-water runoff, and atmospheric sources (burning of fossil fuels) [5]. Therefore, analysis of heavy metals such as Pb and Cr in marine organisms plays an important role in the quality assessment of the marine environment, and they provide useful information for environmental and geochemical research about marine pollution [4].

Chromium is an essential nutrient. It facilities the action of insulin as well as helps to the metabolism and storage of carbohydrate, fat, and protein [6], but excessive level of chromium in marine organisms damages the kidneys, the liver, and blood cells through oxidation reactions, when they are consumed by humans [3, 7]. Lead is a toxic element that can be harmful to marine organisms since they usually show ability to accumulate large amounts of Pb without visible changes in their appearance or yield. In many fishes, Pb accumulation can exceed several times the threshold of maximum level permissible for humans [8].

Many scientists show high pollution levels due to heavy metal toxicity in various fish species making them risky for human consumption [9–12]. Koleleni and Haji [13] reported in their study concentrations ranging from 17.2 *μ*g·g^−1^ for tuna fish to 22.3 *μ*g·g^−1^ for sardine and 0.8 *μ*g·g^−1^ for tuna fish to 1.7 *μ*g·g^−1^ for sardine, for Cr and Pb, respectively. These values were considered too high when compared with the values of the permissible limit for human consumption, as recommended by the Food and Agricultural Organization (FAO; 2004), which indicated that these heavy metals were highly accumulated in the sea port of Zanzibar [13]. A similar study was conducted by Waheed et al. (2017) which founded concentrations between 0.12 *μ*g·g^−1^ and 4.25 *μ*g·g^−1^ for Pb and between 0.58 *μ*g·g^−1^ and 5.11 *μ*g·g^−1^ for Cr in various fish species collected from the Ravi River Pakistan [14].

Measurements of concentrations at the trace level of Pb and Cr play important role in environmental monitoring programs, especially in those focused in the aquatic ecosystem, since it is a critical factor for evaluating potential environment effects because of the associated biotoxicity, high environmental stability, and high occurrence of bioaccumulation in the food chain. Furthermore, one of the most successful ways to obtain valuable information about the interaction of heavy metals with marine organisms is the determination of their concentrations in different seasons of the year, allowing the environmental biomonitoring. Thus, taking into account the above, different analytical techniques have been used with this purpose, such as inductively coupled plasma mass spectrometry (ICP-MS) [15], inductively coupled plasma atomic emission spectroscopy (ICP-AES) [16, 17], graphite furnace-atomic absorption spectrometry (GF-AAS) [18], square wave anodic voltammetry (SWASV) [19], electrothermal atomic absorption spectrometry (ETAAS) [20], hydride generation coupled to atomic absorption spectrometry (HG-AAS) [21], atomic fluorescence spectrometry coupled to the hydride generation (HG-AFS) [22], energy dispersive X-ray fluorescence (EDXRF) [13], and the total reflection X-ray fluorescence (TXRF) spectroscopy [23].

The total reflection X-ray fluorescence spectroscopy is a variant methodology from energy dispersive, including important characteristics such as simultaneous detection of several elements, low detection limit, short measuring times (100–1000 s), and the use of a small sample volume (few mg or *μ*L) [7]. This technique is centered on the incidence of an X-ray beam at a small angle on the flat surface of a support or carrier on which the sample to be analyzed is deposited. In this condition, the scattering effect is minimized and a better peak-background ratio is thus obtained, therefore reducing the detection limits. Multielemental analysis and low cost are two meaningful advantages of this technique [5].

TXRF technique has been used in various areas of science and technology [23]. However, there exist few studies about its use in the analysis of environmental and biological samples, specifically in marine organisms. For this reason, the aim of this work was to develop and validate an accurate, sensitivity, and rapid method for the simultaneous determination of Pb and Cr at trace levels in tissues and fat of marine organisms such as turtle (*Chelonia mydas*), shark (*Rhizoprionodon terraenovae*), and dolphin (*Tursiops truncatus*), utilizing the total reflection X-ray fluorescence (TXRF) spectroscopy.

## 2. Materials and Methods

### 2.1. Chemicals and Reagents

All reagents were of analytical grade. A milli-Q purification system was used to obtain ultrapure water for the preparation of solutions. Glassware was soaked in a 10% nitric acid (HNO_3_, J. T. Baker) solution for 12 hours. Then, the glassware was washed with deionized water and dried. Stock solutions of Pb, Cr, and Ga (1000 mg·L^−1^) were purchased from Fluka and Sigma-Aldrich. The ethylenediaminetetraacetic acid (EDTA, J. T. Baker) was utilized such as chelating agent. A certified reference material, sea lettuce (BCR-279), was obtained from the Community Bureau of Reference of the European Commission (Brussels, Belgium). This material contained zinc, selenium, copper, and lead at high *μ*g·g^−1^ levels. Centrum tablets (contained 35 *μ*g Cr) were purchased from the local market, and this material is a multimineral supplement.

### 2.2. Preparation of Working Solutions

The solution 0.005 mol·L^−1^ EDTA and 1 mol·L^−1^ HNO_3_ was prepared dissolving 0.46 g of EDTA and 17.254 mL of HNO_3_ (14.489 mol·L^−1^) in 250 mL of water.

Working solutions of Pb and Cr were prepared by gradually diluting 1000 mg·L^−1^ of stock solutions in 10 mL of a solution 0.005 mol·L^−1^ EDTA and 1 mol·L^−1^ HNO_3_. In order to correct possible instrument drifts, 20 *μ*L of 10 mg·L^−1^ gallium in nitric acid used as internal standard (IS) was added to obtain a final Ga concentration of 20 *μ*g·L^−1^. In this method, both, the standard and samples, have to be of similar matrix, to produce identical sensitivity, and thus, matrix effects are nullified. Standard solutions and samples were vortexed for 30 s.

### 2.3. Preparation of Sample Solution

Samples of turtle skin (*Chelonia mydas*), shark muscle (*Rhizoprionodon terraenovae*), and dolphin fat (*Tursiops truncatus*) were provided by the Department of Biology of the Veracruz University in Mexico, and they were analyzed using the proposed method. Marine samples were washed in seawater at the sampling site and transferred to the laboratory in small precleaned polyethylene bags under refrigerated conditions. Upon arrival at the laboratory, they were thoroughly rinsed in the deionized water to minimize any possible metal loss. Finally, the samples were frozen and stored (4°C) until analysis.

The wet samples of turtle skin (TORP-033), shark muscle (TIB-15), and dolphin fat (GSD-03) were weighed (0.0253 g, 0.573 g, and 0.029 g, respectively) separately into a 50 mL beaker. Then, nitric acid and hydrogen peroxide were added following the digestion method EPA-3050B [19]. In addition, 1 mL of 0.125 mol·L^−1^ EDTA and 50 *μ*L of 10 mg·L^−1^ gallium used as internal standard (IS) were added, in order to obtain a final concentration of 0.005 mol·L^−1^ and 20 *μ*g·L^−1^ of EDTA and Ga, respectively. Then, mixtures were heated until it dried (80°C). Finally, they were diluted with 25 mL of 1 mol·L^−1^ HNO_3_.

Approximately 0.0222 g of the BCR-279 sample and 0.0495 g of the Centrum tablet sample were weighed separately into a 50 mL beaker. At the same way, the digested procedure was carried out such as marine samples.

### 2.4. Instrumentation and Experimental Procedure

TXRF measurements were carried out by a Bruker S2 Picofox spectrometer (Bruker AXS Microanalysis GmbH, Berlin, Germany), equipped with a Mo tube operating at 600 *μ*A and 50 kV, multilayer monochromator, silicon drift detector (SDD), and energy resolution was 165 eV at 5.9 KeV.

**Figure 1:**
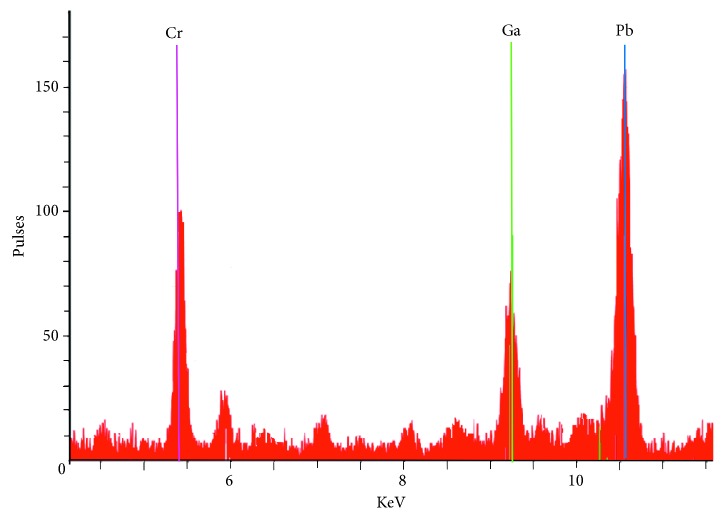
TXRF spectra of Pb, Cr, and Ga in a standard of calibration graph.

Taking care of the precision and using an automatic micropipette, a sample volume of 10 *μ*L was set in the center of a quartz glass sample carrier and dried on a hot plate at 60°C. Three replicates were prepared and measured for each sample. Blank samples were made to identify any possible source of contamination. Subsequently, the samples are placed in a cassette. A maximum of 25 sample carriers within a cassette can be measured automatically. The read time was set in 300 s per sample. The sample frequency was approximately 20 h^−1^. The Spectra software (version 7) was used for data processing and evaluation.  Figure 1 shows typical TXRF spectra of Pb, Cr, and Ga in a standard of the calibration graph. The spectral lines for Pb, Cr, and Ga were *L*_−*α*_ 10.5 KeV, *K*_−*α*_ 5.4 KeV, and *L*_−*α*_ 9.2 KeV, respectively.

After the sample analysis, the quartz glass sample carrier was cleaned following a strict cleaning procedure.

### 2.5. Method Validation

The method was validated as per IUPAC, ICH, and FDA guidelines, and the figures of merit included linearity, range, accuracy, precision, reproducibility, and sensitivity (LOQ and LOD) [24, 25].

#### 2.5.1. Linearity

To evaluate the linearity and range of the method, calibration graphs were constructed when the Pb or Cr peak area/Ga peak area ratio was obtained from the TXRF (*y*-axis) in relation to corresponding concentrations (*x*-axis). The concentration of Pb and Cr in each sample was determined from the respective calibration graph of the element. Three injections from each concentration were analyzed under the same conditions. Linear regression analysis was used to evaluate the linearity of the calibration curve by using the least square linear regression method.

#### 2.5.2. Sensitivity

The limit of detection (LOD) was estimated from three times the standard deviation (SD) of ten replicates of the blank divided by the slope of the calibration curve. The limit of quantification (LOQ) was calculated from ten times the SD of ten replicates of the blank divided by the slope of the calibration curve.

#### 2.5.3. Precision and Reproducibility

The method precision (repeatability) expressed as relative standard deviation (% RSD) was determined by measuring 10 blank sample solutions spiked with the standard solutions of Pb and Cr, each at concentration of 5 *μ*g·L^−1^, under similar conditions (day, analyst, instrument, and sample). Moreover, the reproducibility was estimated from results obtained from the same blank samples which were measured again in five different days.

#### 2.5.4. Accuracy

The accuracy of the assay method was determined by recovery studies. The addition of known concentration standard (20 *μ*g·L^−1^ of Pb and Cr) was employed to analyze the turtle skin (TORP-033), shark muscle (TIB-15), and dolphin fat (GSD-03) samples. Three replicates of each spiked sample were analyzed. In addition, a certified reference material BCR-279 and a Centrum tablet were utilized for the determination of Pb and Cr, respectively. Procedural blanks were always run.

## 3. Results and Discussion

### 3.1. Method Development and Optimization

TXRF operational conditions were obtained from the manufacturer, and chemical properties, such as 1 mol·L^−1^ HNO_3_ and 0.005 mol·L^−1^ EDTA concentration, were obtained from the literature [22, 26]. However, the Ga concentration (20 and 500 *μ*g·L^−1^) was optimized in order to find the best sensitivity, linearity, and wide working concentration range for the simultaneous determination of Pb and Cr. The experiments showed that the Pb peak area/Ga peak area ratio and Cr peak area/Ga peak area ratio were too small if 500 *μ*g·L^−1^ Ga is used; besides, it reduced the working and linear range. Then, 20 *μ*g·L^−1^ Ga was used for further assays.

### 3.2. Method Validation

Table 1 shows figures of merit for the simultaneous determination of Pb and Cr by TXRF method.

#### 3.2.1. Linearity and Working Range

Working range is the range of analytic concentrations over which the method is linear. At the lower end of the concentration range, the limiting factor is LOQ, while at the upper end, limitations are carried out by various effects depending of the instrument response [27].

**Figure 2:**
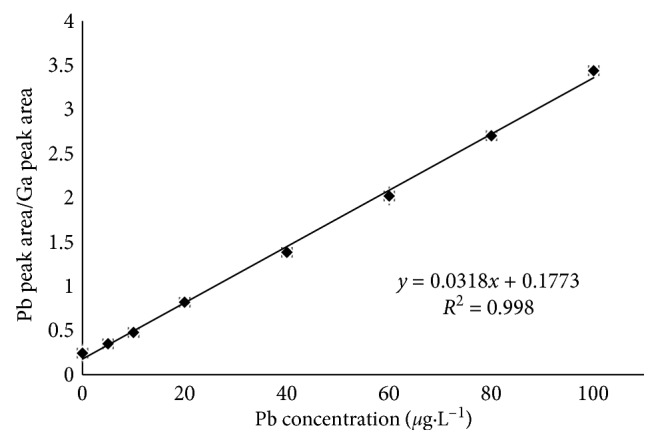
Calibration graph for Pb. Conditions: 0.005 mol·L^−1^ EDTA and 1 mol·L^−1^ HNO_3_. The error bars represent the standard deviation (*n* = 3) for each point.

The results of the linearity study (Figure 2) gave linear relationship over the working range of 5.242 *μ*g·L^−1^ to 100 *μ*g·L^−1^ for Pb, a linear equation was obtained: *y* = 0.0318*x* + 0.1773, and the goodness of fit (*r*^2^) was found to be 0.9980 *n* = 7, indicating a linear relationship between the Pb peak area/Ga peak area ratio and Pb concentrations.

**Figure 3:**
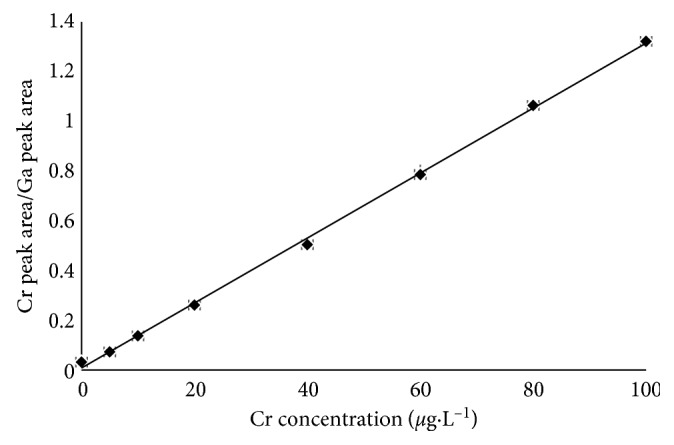
Calibration graph for Cr. Conditions: 0.005 mol·L^−1^ EDTA and 1 mol·L^−1^ HNO_3_. The error bars represent the standard deviation (*n* = 3) for each point.

A calibration graph for Cr (Cr peak area/Ga peak area ratio versus *μ*g·L^−1^ of Cr) with statistically satisfactory fit was obtained (*y* = 0.0131*x* + 0.0084, *r*^2^ = 0.9991, *n* = 7) with a working and linear range from 2.363 *μ*g·L^−1^ to 100 *μ*g·L^−1^ (Figure 3).

#### 3.2.2. Sensitivity

The limit of detection (LOD) is the lowest amount of analyte in a sample that can be detected, but not necessarily quantitated, while the limit of quantification (LOQ) is the lowest amount of analyte in a sample that can be quantitatively determined with suitable precision [24]. Taking into account, the low LODs achieved were 1.573 *μ*g·L^−1^ for Pb and 0.709 *μ*g·L^−1^ for Cr. While, LOQs obtained were 5.242 *μ*g·L^−1^ and 2.363 *μ*g·L^−1^, for Pb and Cr, respectively.

#### 3.2.3. Precision and Reproducibility

In the case of Pb determinations, the precision and reproducibility of the proposed method were 5.1% (*n* = 10) and 3.6% (*n* = 5), respectively, both expressed as RSD. Results obtained for Cr showed a precision value of 4% (*n* = 10) and a reproducibility value of 1.8% (*n* = 5).

#### 3.2.4. Analysis of the BCR-279 and a Centrum Tablet

The accuracy of an analytical procedure expresses the closeness of results obtained by that method to the true value [28]. Then, the accuracy of method was evaluated, analyzing the certified reference material BCR-279 (sea lettuce) and a Centrum tablet (mineral supplement). The obtained results (12.70 ± 0.99 mg·Kg^−1^ of Pb for BCR-279 and 28.47 ± 5.80 mg·Kg^−1^ of Cr for Centrum) were in good agreement with the certified values (13.1 ± 0.40 mg·Kg^−1^ of Pb for BCR-279 and 27.60 ± 0.33 mg·Kg^−1^ of Cr for Centrum). *T*-tests were conducted in order to compare the experimental data with the certified concentrations, and no significant differences were found at a confidence level of 95%, for *n* = 3.

#### 3.2.5. Application to Marine Samples

All samples (TORP-033, TIB-15, and GSD-03) were prepared in 1 mol·L^−1^ HNO_3_ and 0.005 mol·L^−1^ EDTA and were spiked with a known concentration (20 *μ*g·L^−1^) obtained from a corresponding standard. Results showed positive recoveries, over 90% in all cases (Table 2).

The mean results of Pb and Cr concentration were expressed as *μ*g·g^−1^. The turtle skin (TORP-033) sample was 33.8 ± 0.62 *μ*g·g^−1^ Pb and 36.70 ± 0.67 *μ*g·g^−1^ Cr. While the shark muscle (TIB-15) contained 1.4 ± 0.091 *μ*g·g^−1^ Pb and 1.8 ± 0.20 *μ*g·g^−1^ Cr. Finally, the dolphin fat (GSD-03) concentrations were 10.4 ± 2.75 *μ*g·g^−1^ Pb and 45.9 ± 2.33 *μ*g·g^−1^ Cr. The marine samples presented lead and chromium concentrations above the permissible limits of heavy metals concentration in muscle tissues of fishes (2 *μ*g·g^−1^ for Pb and 0.15–1 *μ*g·g^−1^ for Cr) recommended by the Food and Agriculture Organization and World Health Organization (FAO/WHO) guidelines, and these portrait a human health risk through their intake/frequent consumption and thereby their accumulation in the human body [14].

### 3.3. Comparison between the TXRF Method and Other Methodologies for the Simultaneous Determination of Pb and Cr

A brief review of the previously reported methods is given in Table 3. The LODs obtained for the simultaneous determination of Pb and Cr were better in several orders of magnitude than those obtained with a TXRF method proposed by Zarazúa et al. [29], where the yttrium was used as internal standard. In addition, a shorter reading time (300 s) was used by the proposed TXRF method. Besides, our LODs were improved in comparison with the detection limits obtained by the flame atomic absorption spectrometry (FAAS) [18] and energy-dispersive X-ray fluorescence (EDXRF) [13].

The most common internal standard utilized in TXRF methods is the Ga at concentrations levels between 1 and 10 mg·L^−1^ [5, 7]. However, the present TXRF method used 0.0020 mg·L^−1^ (20 ppb) of Ga, which allows it working and linear ranges of 5 × 10^−3^–0.1 mg·L^−1^ and 2 × 10^−3^–0.1 mg·L^−1^ for Pb and Cr, respectively. It should be pointed out that the method for the simultaneous determination of Pb and Cr could be utilized in a wide range of concentrations up to 0.1 mg·L^−1^. In addition, these working and linear ranges were in a good agreement with those obtained with the yttrium (1000 mg·L^−1^) as IS [29].

The sample volume is a great concern, especially in the analysis of environmental and biological samples. The proposed TXRF method allowed using a low sample volume of 10 *μ*L and a sampling frequency of 20 h^−1^. However, the FAAS method requires high sample volume (37.8 mL), since it used preconcentration procedures, reducing at the same time the sampling frequency (18 h^−1^) [30].

The repeatability of the proposed method was 5.1% and 4% for lead and chromium, respectively, expressed as RSD. These values were similar to other procedures where the samples were fish tissues, turtle eggs, algae, and marine sediments [19, 29, 30] but were lower than those obtained by GF-AAS methodology [18].

Furthermore, the consumption of toxic reagents (ammonium pyrrolidine dithiocarbamate, APDC; isobutyl methyl ketone, IBMK) and gases (argon and acetylene) was not necessary in comparison with GF-AAS, FAAS, and SWASV procedures [18, 19, 30].

## 4. Conclusion

Industrial pollution and the discharge of potentially toxic trace metals in aquatic ecosystems cause serious environmental problems such as the metal bioaccumulation in marine organisms. In the present research, a fast, simple, accurate, and sensitivity TXRF method has been developed and validated, for the simultaneous determination of Pb and Cr in samples of marine organisms (turtle skin, shark muscle, and dolphin fat). In addition, the CRM (BCR-297) and a Centrum tablet also were analyzed, with satisfactory results, and hence, it can be employed for a powerful tool in the environmental biomonitoring and toxicological studies. Other advantages offered by the TXRF method are the low detection and quantification limits, the simultaneous determination of Pb and Cr, and a minimum consumption of reagents and sample at the same time that reduces the waste generation.

## Figures and Tables

**Table 1:** Figures of merit for the simultaneous determination of Pb and Cr by the TXRF method.

Analytical parameters	Pb	Cr
Detection limit (*μ*g·L^−1^) (*n* = 10)	1.573	0.709
Quantification limit (*μ*g·L^−1^) (*n* = 10)	5.242	2.363
Regression coefficient *r*^2^ (*n* = 10)	0.998	0.999
Repeatability (RSD %) (*n* = 10, 5 *μ*g·L^−1^)	5.1	4
Reproducibility (RSD %) (*n* = 5, 5 *μ*g·L^−1^)	3.6	1.8
Linear working range (*μ*g·L^−1^)	5.242–100	2.363–100
Sample volume (*μ*L)	10	10
Sample frequency (h^−1^)	20	20

**Table 2:** Simultaneous determination of Pb and Cr in marine samples.

Sample ID	Lead			Chromium		
	Added (*μ*g·L^−1^)	Found (*μ*g·L^−1^)^b^	Recovery (%)	Added (*μ*g·L^−1^)	Found (*μ*g·L^−1^)^b^	Recovery (%)
TOP-033^a^	0	34.20 ± 1.50	—	0	37.10 ± 0.012	—
	20	53.80 ± 0.07	98	20	57.20 ± 0.09	100

TIB-15^a^	0	32.40 ± 0.07	—	0	40.50 ± 0.20	—
	20	51.30 ± 0.10	95	20	60.70 ± 0.20	101

GSD-03^a^	0	12.10 ± 0.06	—	0	53.30 ± 0.05	—
	20	31.20 ± 0.10	96	20	71.30 ± 0.02	90

^a^The results are reported as the Pb and Cr concentration in the analyzed solutions. ^b^The results are expressed as the mean value ± SD (*n* = 3).

**Table 3:** Comparison between the TXRF methodology and other methodologies for the simultaneous determination of Pb and Cr.

Detection system^a^	LOD (mg·L^−1^)		Linear working range (mg·L^−1^)		Sample volume (mL)	RSD (%)		Sample introduction	Type of sample	Ref.
	Pb	Cr	Pb	Cr		Pb	Cr			
TXRF (Ga, IS)	1.573 × 10^−3^	7.09 × 10^−4^	5 × 10^−3^–0.1	2 × 10^−3^–0.1	0.01	5.1	4	Liquid	Turtle skin, shark muscle, and dolphin fat	Present work
TXRF (Y, IS)	0.03	0.05	0.2–1.9	1.1–9.3	0.02	3	5	Liquid	Fish	[29]
EDXRF	0.5	5.2	0.6–7.4	16.1–20.7	—	3	3	Pellets	Fish	[13]
GF-AAS	0.0208	0.0171	10–50	1–13	0.015	6.2	7.9	Liquid	Fish	[18]
SWASV	0.031	—	10–30	—	5	5	—	Liquid	Algae and turtle eggs	[19]
FAAS	—	8 × 10^−4^	—	1 × 10^−3^–0.04	37.8	—	3.2	Liquid	Marine sediments	[30]

^a^TXRF, total reflection X-ray fluorescence; EDXRF, energy dispersive X-ray fluorescence; IS, internal standard; GF-AAS, graphite furnace-atomic absorption spectrometry; SWASV, square wave anodic stripping voltammetry coupled to disposable screen-printed electrodes; FAAS, flame atomic absorption spectrometry.

## Data Availability

The data used to support the findings of this study are available from the corresponding author (bbeltran@uach.mx) upon request.
